# Acute amyloid-β exposure disrupts insulin signaling in blood–brain barrier endothelial cell culture models

**DOI:** 10.21203/rs.3.rs-8436616/v1

**Published:** 2026-01-12

**Authors:** Douglas A. Nelson, Suresh K Swaminathan, Hannah S. Seo, Samira M. Azarin, Krishna R. Kalari, Karunya K. Kandimalla

**Affiliations:** University of Minnesota; University of Minnesota; University of Minnesota; University of Minnesota; Mayo Clinic; University of Minnesota

**Keywords:** blood–brain barrier, Alzheimer’s disease, insulin signaling, amyloid-β, endothelial cells, Akt signaling, reverse-phase protein array

## Abstract

**Background:**

Brain insulin resistance and cerebrovascular dysfunction emerge early in late-onset Alzheimer’s disease, but how amyloid-β (Aβ) disrupts insulin signaling at the cerebrovascular blood–brain barrier—a major site of insulin receptor signaling and transport into the brain—remains unclear.

**Methods:**

We exposed two distinct human blood-brain-barrier endothelial cell models to soluble Aβ40 or Aβ42 for 1 h, followed by 100 nM insulin for 10 min. Protein and phosphoprotein responses were quantified by reverse-phase protein array, and differential expression was evaluated using linear models.

**Results:**

Aβ40 reduced insulin-stimulated Akt activation and converted insulin’s normal inhibition of AMPK into modest stimulation. Aβ42 did not alter insulin-stimulated Akt signaling but moderately suppressed basal Akt activation.

**Conclusions:**

These findings suggest that Aβ40 acutely impairs insulin signal transduction in BBB endothelial cells, supporting a model in which vascular Aβ exposure contributes directly to the early development of brain insulin resistance in AD.

## Background

Alzheimer’s disease (AD) is a multifaceted neurodegenerative disorder characterized by the accumulation of amyloid-β (Aβ) peptides and neurofibrillary tangles of hyperphosphorylated tau (1-4). In late-onset AD, these Aβ and tau pathologies consistently co-occur with cerebrovascular dysfunction and brain insulin resistance, both emerging early in the disease process(5-7).

Among the cerebrovascular changes associated with late-onset AD, dysfunction of the blood–brain barrier (BBB) is especially important. The BBB forms the primary interface between blood and brain, regulating nutrient delivery and clearance of metabolic waste, including toxic Aβ peptides (8). The BBB is an integral component of the neurovascular unit (NVU), an interdependent association of neurons, astrocytes, pericytes, and cerebral endothelium that couples neuronal activity to local changes in cerebral blood flow (9). In AD, the BBB and broader NVU exhibit early and progressive dysfunction (10).

Metabolic syndrome and type 2 diabetes are major risk factors for AD (11). Chronic peripheral insulin resistance disrupts metabolic insulin signaling, leading to impaired energy metabolism and elevated inflammation in the brain that accelerates AD progression (12). Notably, insulin receptors are enriched in brain microvascular endothelial cells compared to other NVU components, positioning the BBB endothelium as the primary site for insulin entry and signaling within the NVU (13). Impaired insulin signaling at the BBB is thought to disrupt neurovascular coupling and significantly reduce Aβ clearance from the brain (9, 12, 14). These findings suggest a positive feedback loop: Aβ impairs endothelial insulin signaling, which further limits Aβ clearance (12, 14). Understanding how Aβ exposure and insulin resistance drive BBB dysfunction at the molecular level is therefore critical.

The two most abundant Aβ isoforms, Aβ40 and Aβ42, are characterized by distinct pathological distributions: Aβ40 predominates in vascular amyloid deposits associated with cerebral amyloid angiopathy, while Aβ42, although less abundant overall, is the dominant component of parenchymal plaques (15, 16). The two isoforms also exert distinct effects on the cerebrovascular endothelium, but the mechanisms and isoform specificity of Aβ interference with insulin signaling remain poorly defined (14, 17).

Prior studies show that Aβ can impair insulin signaling in both neurons and BBB endothelial cells, but the mechanistic basis for this disruption—and whether Aβ40 and Aβ42 differ in their effects on BBB insulin transduction—remains unknown (14, 17). To characterize the direct effects of Aβ exposure, we examined the acute response to Aβ40 and Aβ42 on BBB insulin signaling. Identifying these transient molecular changes is essential because acute pathway perturbations often reveal the mechanistic entry points that lead to degenerative processes, even though the chronic phenotypes may differ markedly (18-21). Accordingly, these early signaling changes can provide insight into how chronic endothelial dysfunction and brain insulin resistance can emerge. To capture these acute responses within a broader signaling context, we utilized reverse-phase protein arrays (RPPA), which enable parallel measurement of hundreds of proteins and phosphoproteins. This study, therefore, defines how acute exposure to Aβ40 and Aβ42 perturbs BBB endothelial insulin signaling, establishing the mechanistic context needed to evaluate isoform-specific contributions to vascular insulin resistance.

## Methods

### Aβ and insulin Peptides

Aβ40 and Aβ42 were obtained from Aapptec (Louisville, KY). Soluble Aβ40 and Aβ42 solutions were prepared according to the procedure described by Klien and characterized as described in our earlier publications (22). Briefly, Aβ films were formed after dissolving the lyophilized peptide in HFIP and evaporating the solvent under vacuum. The resultant films were hydrated by sequential addition of 50 μL DMSO, 50 μL deionized water, and 100 μL pH 8.3 F12 medium, with brief sonication after each step. Then the solutions were centrifuged at 10,000 rpm for 5 min at 4°C to remove any insoluble aggregates, and the supernatant was diluted in cell culture medium to final concentration of 1 μM, as assayed by ELISA. Vehicle controls were prepared using the same DMSO, water, and medium sequence without Aβ peptide.

Insulin powder (Sigma, Saint Louis, MO) was solubilized in 0.1 N sodium carbonate (5.3 mg/mL). A 1000 μM master stock was generated at 5.808 mg/mL and stored at 4°C. From this, a final concentration of 100 nM was prepared in the cell culture medium, and the cells were treated with 100 nM insulin for 10 min.

### Cell cultures

Human cerebral microvascular endothelial cell (hCMEC/D3) line was a generous gift from Pierre-Olivier Couraud of the Institut Cochin (Paris, France). The endothelial cells were cultured according to the protocols provided by the Couraud group (23). Polarized hCMEC/D3 cell monolayer were cultured by seeding the cells at passage 34 on 24 mm Transwells as detailed in our earlier publications (24).

Brain-specific microvascular endothelial-like cells (iBMECs) were differentiated from IMR90-4 (WiCell, Madison, WI) human induced pluripotent stem cells (hiPSC), according to the procedures reported by Stebbins et al. (25). On day 8, iBMECs were transferred to collagen IV/fibronectin (CN IV-FN)–coated 24.6 mm CellTreat^®^ inserts (CELLTREAT Scientific Products, Pepperell, MA) at a concentration of 1x106 cells/cm2. The iBMECs were cultured in endothelial medium consisting of Human Endothelial Serum-Free Medium (Thermo Fisher Scientific) supplemented with 1% platelet-poor plasma derived serum (PDS; Biomedical Technologies), 20 ng/mL basic fibroblast growth factor (PeproTech), and 10 μM retinoic acid (Millipore Sigma). On day 9, cells were maintained in Human Endothelial Serum-Free Medium containing only 1% PDS.

For coculturing, frozen human brain vascular pericytes (hBVPs; ScienCell, Carlsbad, CA) were thawed and expanded in poly-D-lysine (PDL)-coated T-75 flasks using Dulbecco’s Modified Eagle’s Medium (DMEM, Millipore Sigma) supplemented with 10% fetal bovine serum (FBS; Thermo Fisher Scientific). Human astrocytes (HA; ScienCell, catalog #1800) were expanded per vendor instructions in human astrocyte growth medium (Cell Applications, Inc.) on PDL-coated plates. Pericytes and astrocytes were then seeded at a 1:1 ratio by cell number onto the bottoms of PDL-coated 6-well plates at a total cell density of 312,500 cells per well in iBMEC medium. After iBMECs formed a barrier, the inserts were placed into the wells containing pericytes and astrocytes to establish a non-contact BBB model; cultures were maintained at 37°C in a humidified 5% CO_2_ atmosphere.

### Treatments with Aβ and insulin

Treatments and lysate preparation for iBMECs and hCMEC/D3 cells were the same. At the end of each treatment, the spent medium was removed, and cells were washed twice with DPBS. Then DMEM (2.5 mL) was added to the abluminal side. One milliliter of either control medium or Aβ (1 μM) was added luminally and incubated for 1 h. Where indicated, cells were spiked with 100 nM insulin for the last 10 min before the termination of the experiment. All Aβ and insulin incubations were carried out on the luminal side.

Treatment groups included untreated medium, vehicle, insulin (10 min), Aβ40 (1 h), Aβ42 (1 h), Aβ40 (1 h) plus insulin, and Aβ42 (1 h) plus insulin. Each of the seven conditions had three replicates, totaling 21 wells across seven treatments. Plates 1 (untreated) and 2 (insulin) were processed in tandem; followed by Plates 3 (Aβ40 alone) and 4 (Aβ40 plus insulin); Plates 5 (Aβ42 alone) and 6 (Aβ42 plus insulin); and plate 7 (vehicle only). These groupings defined four independent RPPA runs (batches).

At the end of treatment, plates were placed on ice. Cells were immediately washed once with ice-cold HBSS, followed by the addition of ice-cold HBSS containing protease and phosphatase inhibitors. Then RIPA lysis buffer containing protease and phosphatase inhibitors (50 μL per well) was added. Cells were scraped with a cell scraper and collected in pre-chilled microcentrifuge tubes. This sequence was repeated for all wells. Lysates were sonicated for 4 min until clear and centrifuged at 10,000 rpm for 10 min at 4°C. The supernatants were transferred to fresh 0.5 mL tubes, pellets discarded, and samples stored at − 20°C. For final preparation, 4X SDS without bromophenol blue and with beta-mercaptoethanol was added, samples were boiled for 5 min, and stored at − 80°C.

### Reverse Phase Protein Array (RPPA) assays

RPPA was performed by the MD Anderson Functional Proteomics RPPA Core (Set151; 21 samples; 306 antibodies). Lysates were blotted as five two-fold dilutions (undiluted, 1:2, 1:4, 1:8, 1:16) on nitrocellulose slides. Then the blots were probed using tyramide amplification, developed with DAB, scanned on a Huron TissueScope, and quantified in Array-Pro. Relative protein levels for each antibody were estimated by SuperCurve interpolation of the five-point dilution series. The Core provided sample-specific loading correction factors CF1 and CF2, where CF1 is calculated within the submitted set and CF2 across all samples printed on the slide; samples with CF2 < 0.25 or > 2.5 are flagged as protein-loading outliers recommended for exclusion. The hCMEC/D3 samples had CF1 0.83–1.35 and CF2 0.45–0.72, whereas iBMEC samples had CF1 0.66–1.33 and CF2 0.56–1.10. None of the samples met CF2 outlier criterion and were not excluded.

### RPPA data analysis

Sample-wise median centering was applied after inspection of raw expression distributions and variance profiles across samples. Probes corresponding to proteins that are not biologically relevant to the BBB endothelium were excluded. The exclusion list was curated from established literature and cross-referenced for lack of functional abundance based on median values from pseudobulk analysis of a public transcriptomic dataset (NCBI GEO: GSE163577). A list of filtered probes is provided in Supplementary List 1. A total of 268 probes were included after filtering, out of which 62 were phosphoprobes. Three filtered probes were relevant to pericyte biology and were not removed from the pericyte/astrocyte analysis.

Principal component analysis (PCA) was performed for each two-group contrast to assess replicate clustering before differential expression analysis. Cluster coherence was quantified using silhouette widths computed on the principal components explaining ≥ 95% of the variance, and Mahalanobis distances in two dimensions using a pooled within-class covariance estimate.

To reduce the influence of high-variance and outlier probes, differential expression was fit with Limma using empirical-Bayes moderation configured with a mean–variance trend (trend = TRUE) to accommodate RPPA’s intensity-dependent variance and robust hyperparameter estimation (robust = TRUE). We estimated sample precision weights with arrayWeights for each affected contrast. Benjamini-Hochberg multiple hypothesis correction was applied per contrast.

To generate composite scores that emphasize probes supported by both meaningful effect size and statistical evidence, we transformed differential expression statistics into bounded scores using raised-cosine functions with log_2_-fold change (LFC) parameters log2(1.1) and log2(1.5), and FDR parameters − log10(0.10) and − log10(0.001). Composite scores were then calculated as the geometric mean, penalizing imbalance between the two components.

To evaluate inter-batch shifts, we defined a set of invariant total-protein probes based on within-batch comparisons. A probe›› was considered invariant if, within each of the three two-group batches (untreated vs insulin, Aβ40 vs Aβ40 + insulin, Aβ42 vs Aβ42 + insulin), the estimated log_2_ fold-change between groups is ± 0.1 within a 95% confidence interval. Confidence intervals were calculated from the Limma model using empirical Bayes-moderated standard errors. Only probes meeting this criterion in all three batches were retained. Probes with any plausible mechanism for acute Aβ sensitivity were excluded from the invariant set. For each inter-batch comparison, we summarized the distribution of differences across the invariant set by reporting the Wilcoxon signed-rank p-value, the median log_2_ fold-change, and the median absolute deviation (MAD).

## Results

### Differential expression overview and quality control

We used reverse-phase protein arrays (RPPA) to quantify protein and phosphoprotein abundance using 268 antibody probes in two BBB endothelial models. Cells were exposed to vehicle, Aβ40, or Aβ42 (1 μM, 1 h), with or without insulin (100 nM, 10 min). Each condition included three biological replicates, run across four RPPA batches. We first established quality control metrics, then examined Aβ effects without insulin, validated insulin responses in both models, and finally assessed how Aβ modulates insulin-stimulated signaling.

We assessed replicate quality and treatment separation using principal component analysis. hCMEC/D3 samples exposed to Aβ plus insulin showed reduced within-group coherence and increased variability (Supplementary Table 1), so we applied sample-specific precision weights in the differential expression analysis for these contrasts. Table 1 summarizes the number of significantly changed proteins (FDR < 0.05) across eight key comparisons. Aβ40 consistently produced differential expression in more proteins than Aβ42 in corresponding contrasts across iBMECs and in non-insulin comparisons for hCMEC/D3. The hCMEC/D3 vehicle-untreated comparison showed substantially elevated differential expression (103 probes), consistent with the batch effect identified in the vehicle run.

**Figure 1:**
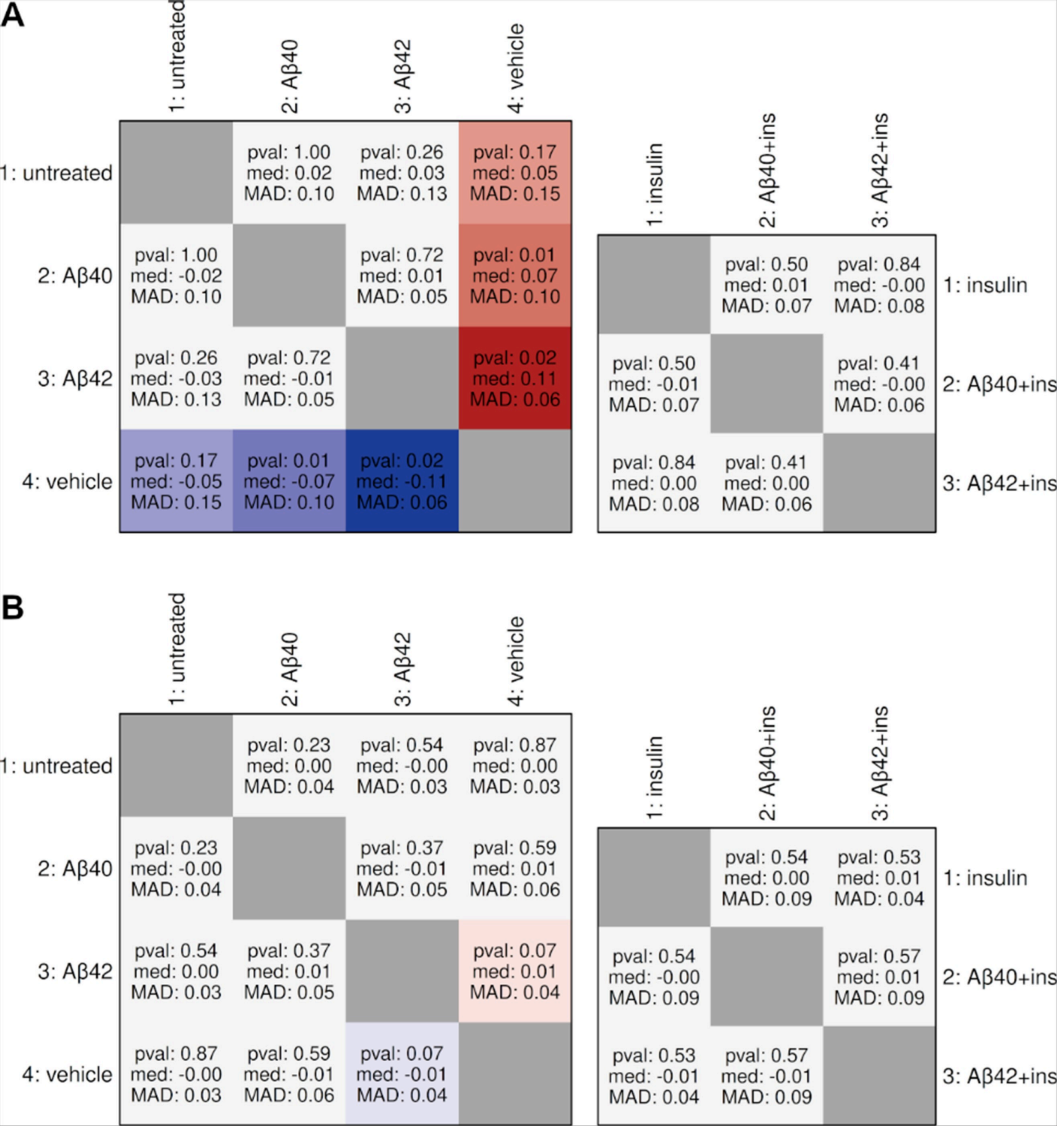
Evaluation of inter-batch shifts using invariant total-protein probes. RPPA analyses were run in four batches: (1) untreated and insulin, (2) Aβ40 and Aβ40 with insulin, (3) Aβ42 and Aβ42 with insulin, and (4) vehicle alone. Invariant probes were defined as total-protein probes with 95% confidence intervals for log_2_ fold-change within ±0.1 in all three two-group batches. This intra-batch invariant set was used to assess batch-to-batch consistency. A (top) shows hCMEC/D3 results: the left matrix is a 4x4 comparison of all pairwise inter-batch shifts among the non-insulin groups (untreated, Aβ40, Aβ42, vehicle), and the right matrix is a 3x3 comparison among the insulin-containing groups (insulin, Aβ40 with insulin, Aβ42 with insulin). B (bottom) shows the corresponding iBMEC results with the identical structure. Each cell reports three statistics (top-to-bottom): Wilcoxon signed-rank p-value (pval), median log_2_ fold-change (med), and median absolute deviation (MAD). Cells are colored when pval < 0.20 and ∣med∣ ≥ 0.01. Red indicates positive shifts and blue indicates negative shifts, defined as the row group minus the column group; color intensity scales with the magnitude of the median shift. Diagonal cells represent comparisons of a batch to itself and are shaded gray.

To assess residual batch effects after normalization, we identified invariant total-protein probes showing stable abundance within batches and examined their consistency across batches (Fig. 1). Twelve invariant probes were identified in hCMEC/D3 and 53 in iBMECs. Inter-batch comparisons showed general stability in both models, with the exception of the hCMEC/D3 vehicle batch, which exhibited systematic negative displacement.

**Figure 2:**
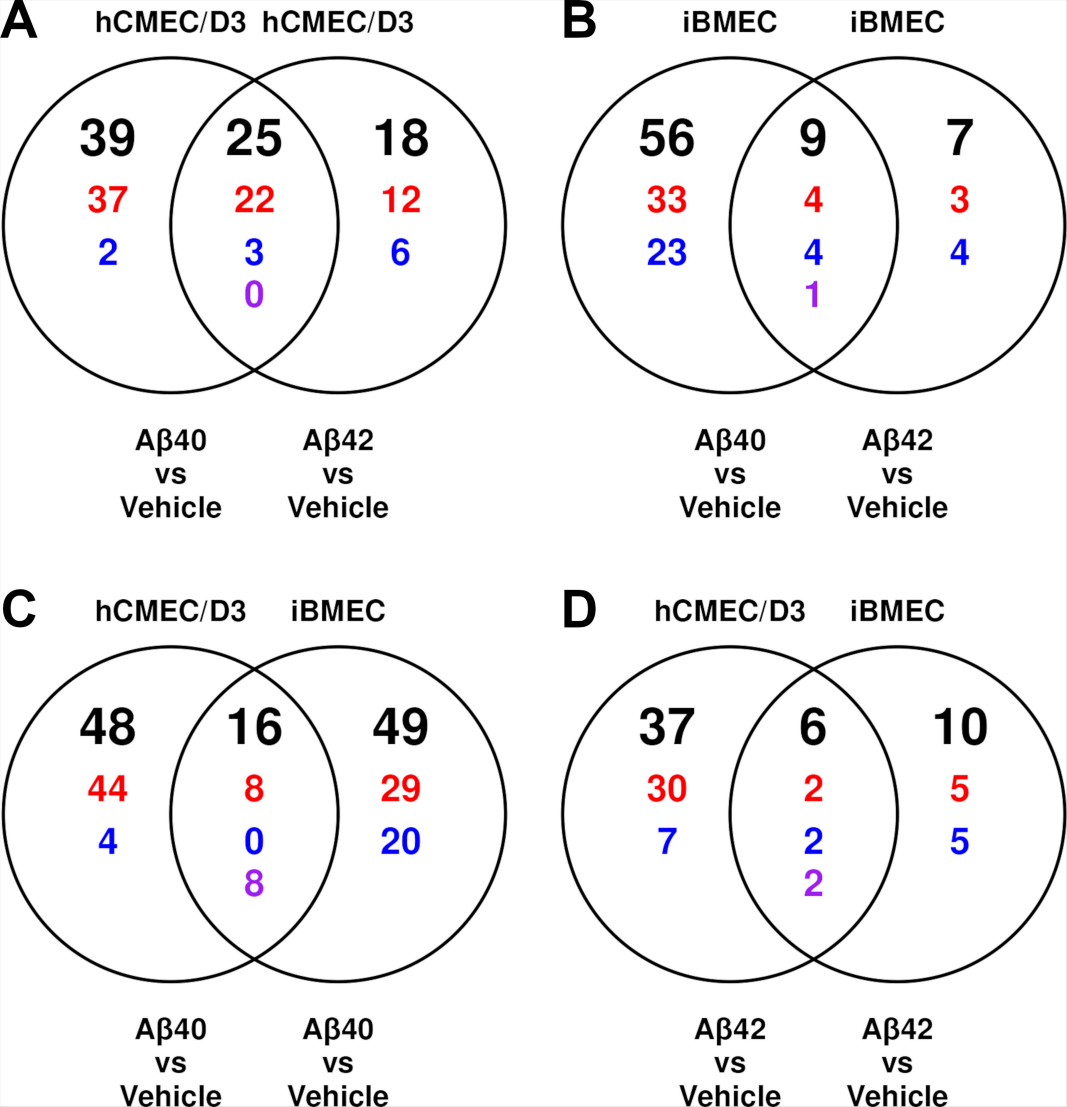
Overlap of differentially expressed proteins between Aβ isoforms and between BBB endothelial models. Differential expression is defined by FDR < 0.05, with direction based on log_2_ fold-change sign. Black numbers above each diagram indicate total differentially expressed proteins. Colored numbers denote directional overlap: red for up/up, blue for down/down, and purple for opposite-sign overlaps. Top: Aβ isoform overlaps for Aβ-vehicle contrasts in hCMEC/D3 (**A**) and iBMECs (**B**). Bottom: cell model overlaps for Aβ-vehicle contrasts with Aβ40 (**C**) and Aβ42 (**D**).

Venn overlap analysis of DE probes (Fig. 2) shows that, within each endothelial model, the two Aβ isoforms exhibited modest overlap: at least half of Aβ42-responsive probes were also differentially expressed in the same direction under Aβ40 in both models. Concordance between the two BBB models was lower, with only one-eighth (Aβ40) and one-quarter (Aβ42) of iBMEC responses replicated in the hCMEC/D3 model.

### Aβ effects without insulin

**Figure 3:**
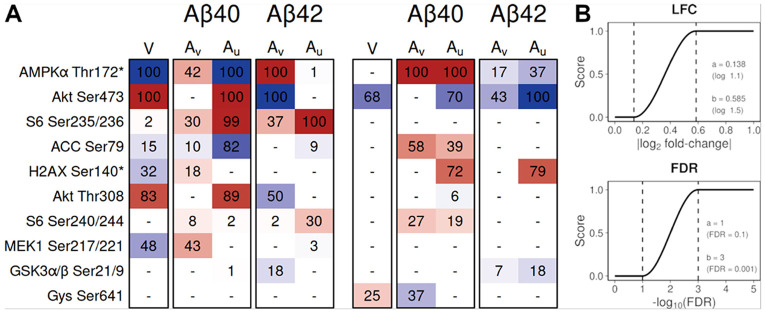
Differentially expressed phosphoprotein probes in BBB endothelial models after one-hour Aβ exposure. **A:** Heatmap matrices show differentially expressed phosphoproteins in hCMEC/D3 (left) and iBMEC (right). Each model shows the vehicle – untreated contrast (V) and, for each Aβ isoform, Aβ – vehicle (Av) and Aβ – Untreated (Au). Each cell contains a composite score computed as the geometric mean of two raised cosine functions—one based on ∣log_2_ fold-change∣ (LFC) and one on −log_10_(FDR)—each scaled to [0, 1], with final scores multiplied by 100. Red indicates upregulation and blue indicates downregulation. Only the ten highest-scoring probes are shown. Antibody probes marked with an asterisk (*) were not validated by the MD Anderson RPPA Core. **B:** Raised cosine scoring functions. For LFC (top), the transition occurs between a = 0.138 (log_2_ 1.1) and b = 0.585 (log_2_ 1.5). For FDR (bottom), the transition occurs between a = 1 (FDR = 0.1) and b = 3 (FDR = 0.001).

Before examining how Aβ modulates insulin signaling, we first characterized Aβ effects in the absence of insulin to establish baseline responses. Figure 3 summarizes the top phosphoprotein expression changes in response to one-hour amyloid exposure in both BBB endothelial models. The score matrix integrates fold-change and FDR into a single metric reflecting magnitude and statistical reliability. Vehicle exposure upregulated Akt Ser473 and Akt Thr308 relative to untreated controls, opposite to the direction of vehicle batch bias. In the hCMEC/D3 model, Aβ42 reversed this upregulation. For other phosphoproteins, we focused on those showing consistent directional changes in both the Aβ – vehicle and Aβ – untreated contrasts. In hCMEC/D3 cells, both Aβ isoforms upregulated S6 Ser235/236 and S6 Ser240/244, while Aβ40 reduced ACC Ser79. In iBMECs, Aβ40 upregulated AMPKα Thr172, ACC Ser79, and S6 Ser240/244, whereas Aβ42 downregulated AMPKα Thr172, Akt Ser473, and GSK3α/β Ser21/9.

### Insulin signaling in hCMEC/D3 and iBMEC models

**Figure 4:**
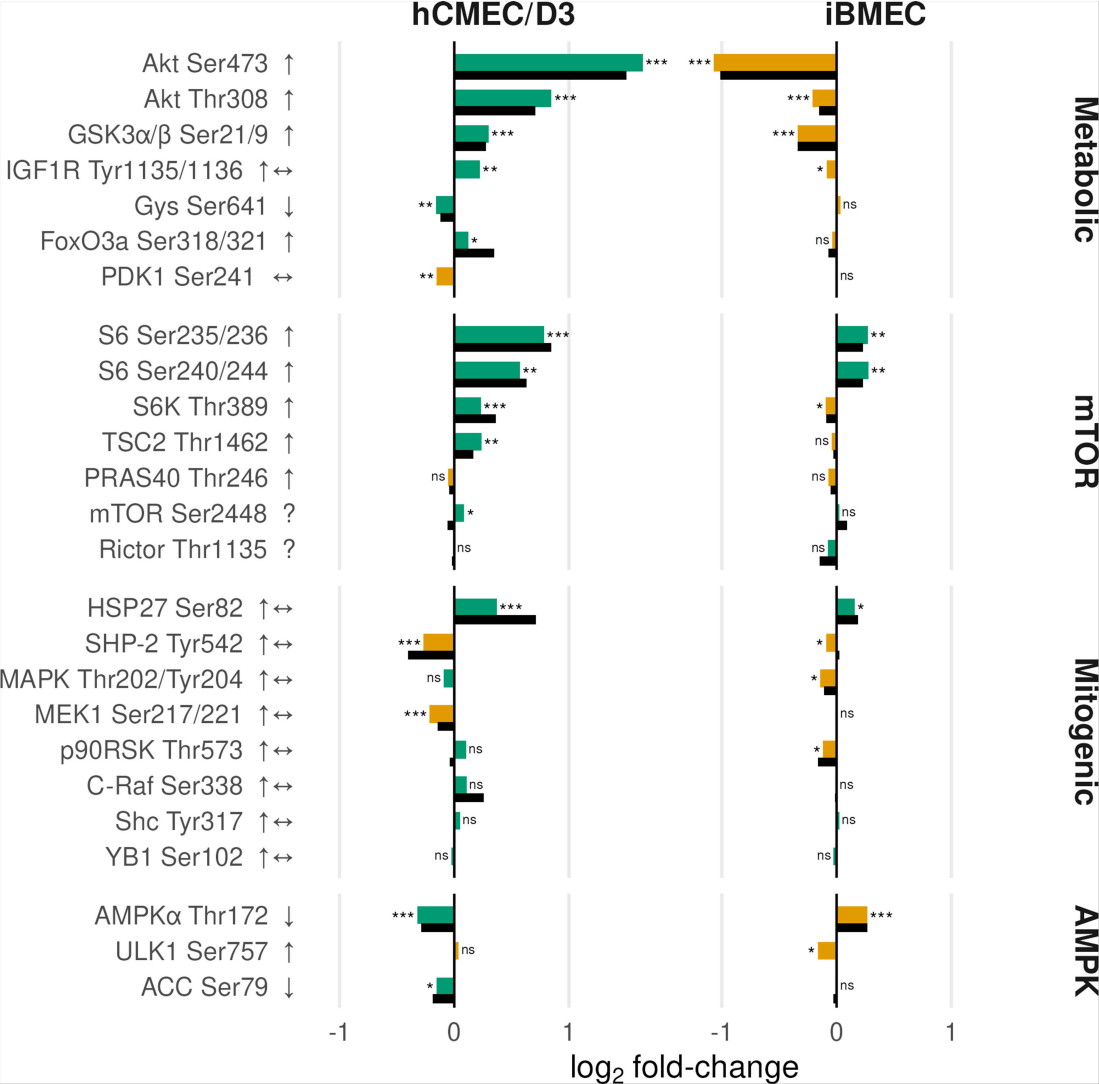
Log_2_-fold changes in insulin signaling probes in hCMEC/D3 and iBMEC BBB models. Cells were treated with 100 nM insulin for 10 minutes and compared with untreated controls. Probes are grouped into four functional pathways: metabolic, mTOR, mitogenic, and AMPK. Expected direction of response is shown to the right of each probe (↑ increase, ↓ decrease, ↔ stable, ? no expectation). Colored bars represent observed log_2_ fold-change: green indicates consistency with expectation, yellow/orange indicates divergence. Asterisks denote statistical significance (*, **, *** for FDR < 0.05, < 0.01, < 0.001; ns = not significant). Black bars represent phospho-to-total protein ratios. Probes lacking black bars have no corresponding total-protein measurement (IGF1R Tyr1135/1136, ULK1 Ser757, Shc Tyr317, YB1 Ser102). For PDK1 Ser241, the phospho-to-total ratio is near unity (zero on log scale).

Proteins with differential phosphorylation after 10-minute exposure to 100 nM insulin in both BBB endothelial models is shown in Fig. 4. These phosphoproteins are organized into four functional pathway groups: metabolic (Akt and immediate substrates), mTOR-centered (S6/S6K/TSC2/PRAS40/mTOR/Rictor), mitogenic (MEK-ERK cascade), and AMPK. Results are shown as log_2_ fold-changes for phosphoproteins, with phospho/total protein ratios displayed where corresponding total protein measurements were available. Phospho/total protein ratios were generally consistent with phosphoprotein changes alone, with the exception of PDK1 Ser241, where the ratio remained near unity as expected given its constitutive phosphorylation. The hCMEC/D3 model exhibited canonical insulin responses across metabolic and mTORC1 pathways. Akt Thr308 and Akt Ser473 showed the strongest activation, with corresponding increases in downstream mTOR-axis substrates (S6 Ser235/236, S6K Thr389, and TSC2 Thr1462). AMPKα Thr172 and ACC Ser79 decreased, consistent with insulin-mediated AMPK suppression. In contrast, iBMECs showed non-canonical insulin responses, with key metabolic nodes such as Akt Thr308 and GSK3α/β Ser21/9 changing opposite to expected directions. Subsequent analyses of Aβ effects on insulin signaling therefore focus exclusively on the hCMEC/D3 model.

### Aβ effects on insulin signaling

**Figure 5:**
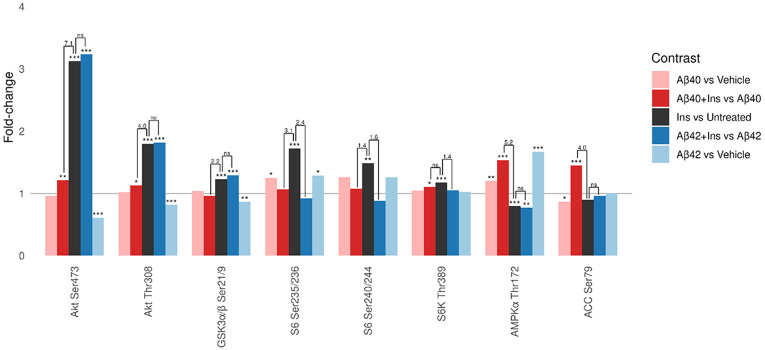
Insulin-responsive probes in hCMEC/D3 BBB endothelial cells with and without Aβ exposure. Shown are selected metabolic, mTOR, and AMPK probes with significant insulin responses. Insulin effects are shown in untreated cells and in cells exposed to Aβ40 or Aβ42 (both with DMSO vehicle). For reference, Aβ – vehicle contrasts without insulin are also shown. The horizontal line at fold-change = 1 denotes no change (log_2_ fold-change = 0) within each contrast. Asterisks (*, **, ***) denote FDR < 0.05, < 0.01, and < 0.001, respectively; ns = not significant. Numeric annotations above comparison brackets indicate −log_10_(FDR) from second-order contrasts, each bracket denoting the significance of differences between the effect of insulin on untreated cells and its effect on Aβ-treated cells.

**Figure 6:**
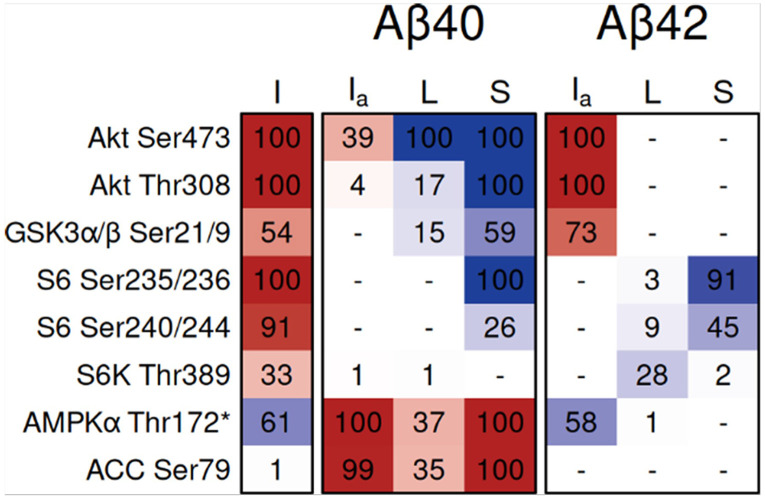
Differentially expressed insulin-responsive phosphoproteins in hCMEC/D3 with Aβ40 and Aβ42 exposure. Contrasts: I (insulin – untreated), I_a_ (insulin with Aβ – Aβ), L (level difference: insulin with Aβ – insulin), and S (second-order contrast measuring the shift in insulin response: I_a_ – I). Each cell contains a score computed as the geometric mean of two functions—one based on log_2_ fold-change and one on FDR—each scaled to [0, 1]. Final scores are multiplied by 100. Scoring function plots are provided in Supplementary Figure 1. Red indicates upregulation and blue indicates downregulation. Antibody probes marked with an asterisk (*) were not validated by the MD Anderson RPPA Core.

To quantify how Aβ modulates insulin signaling in hCMEC/D3 cells, we examined insulin responses in the presence and absence of Aβ exposure. We used second-order contrasts [(Aβ with insulin – Aβ) – (insulin – untreated)] to measure whether Aβ amplifies, attenuates, or reverses insulin-stimulated changes at each phosphoprotein node. Figure 5 shows fold-changes for five contrasts involving Aβ and insulin effects, with statistical comparisons showing the second-order contrasts. To distinguish between attenuation, augmentation, reversal, and ambiguous outcomes, we integrated the second-order comparison with three other insulin-related contrasts (Fig. 6) using decision rules formalized in Supplementary Fig. 1. Table 2 summarizes the integrated interpretation for key insulin-responsive phosphoproteins.

Aβ40 produced the most substantial alterations in insulin signaling. Within the metabolic pathway, Aβ40 markedly attenuated insulin-stimulated Akt activation at both Ser473 and Thr308, with corresponding attenuation of its downstream target GSK3α/β Ser21/9. In the AMPK pathway, Aβ40 reversed insulin’s suppression of AMPKα Thr172 and its direct substrate ACC Ser79, instead producing net stimulation. Both Aβ40 and Aβ42 reduced insulin-stimulated phosphorylation of S6 Ser235/236 and S6 Ser240/244, with Aβ42 also reducing S6K Thr389.

## Discussion

### Broad mechanisms of BBB insulin resistance

BBB insulin resistance in late-onset AD has been attributed to reduced insulin receptor abundance, inhibitory serine phosphorylation of IRS proteins, and direct Aβ interaction with the insulin receptor (INSR). Reductions in INSR abundance have been linked to BACE1-dependent receptor degradation, which occurs over longer timescales than examined in this study (26). Consistent with this, total INSR protein levels were unchanged at 1 h. While acute signaling changes are not consequences of receptor depletion, altered receptor localization or availability remain possible. We therefore consider two general insulin resistance mechanisms: (1) inhibitory serine phosphorylation of IRS proteins driven by kinase pathways engaged during Aβ exposure, and (2) direct Aβ-INSR interaction, which could alter receptor availability or INSR signal output.

Aβ is known to induce oxidative stress, particularly through direct engagement with the RAGE receptor, which activates NADPH oxidase and other ROS-generating pathways (27). Resulting oxidative stress can activate stress kinases (JNK1/2, IKKβ, PKCβII) that phosphorylate IRS at inhibitory serine sites (28, 29). In parallel, MAPK-RSK and p70S6K (S6K) can phosphorylate IRS independently of oxidative stress (28, 30). Evidence for IRS serine phosphorylation is inconclusive, however, as functional IRS probes were absent, and the measured phosphorylating kinases (JNK1, PKCβII, MAPK, S6K) showed no activation at 1 h, although activation at earlier time points cannot be excluded.

Experiments using human placental membranes and purified insulin receptor preparations showed that Aβ40 and Aβ42 reduce insulin binding and inhibit insulin-stimulated receptor autophosphorylation in a manner consistent with competitive inhibition (31). Both isoforms engage the insulin-binding ectodomain with relatively low affinity, but within relevant concentrations (31). Structural modeling of monomeric Aβ40 also supports an energetically favorable but weaker interaction at the receptor ectodomain (32). Lower affinity binding does not simply produce weaker responses; for example, IGF1 and insulin elicit qualitatively distinct signaling profiles despite binding the same receptor (33). INSR activation involves coordinated conformational changes and multisite autophosphorylation that determine downstream signaling branch balance. Aβ binding to INSR therefore likely induces a distinct or incomplete signaling state rather than mimicking insulin action at lower potency, though downstream consequences remain poorly characterized.

### Aβ-only responses under batch effects

Given RPPA's semi-quantitative nature, our analytical approach—including invariant-probe batch assessment, dual-contrast validation, and composite scoring—prioritized identifying robust qualitative patterns for targeted validation over maximizing discovery. The invariant probe set analysis (Fig. 1) revealed systematic negative displacement in the hCMEC/D3 vehicle batch. This bias affects interpretation of vehicle-related contrasts: proteins appearing downregulated in vehicle-untreated or upregulated in Aβ – vehicle comparisons are most susceptible to confounding. However, several patterns oppose this bias direction or are additionally supported by non-vehicle contrasts and therefore likely represent biological effects rather than technical artifacts.

The stronger response to Aβ40 compared to Aβ42 treatment in hCMEC/D3 cells cannot be attributed to systemic batch shift, since the pattern appears in both cell models and both contrasts share the same vehicle reference (Fig. 2 and Fig. 3). This pattern aligns with Aβ40’s greater vasculotropic character (34). Second, phosphorylation of Akt at S473 and T308 is robustly increased in the vehicle – untreated comparison, which is opposite in direction to the vehicle batch shift (Fig. 3) and consistent with reports of DMSO-induced Akt activation (35).

To identify robust patterns, we focused on phosphoproteins showing consistent directional changes in both Aβ – vehicle and Aβ – untreated comparisons (Fig. 3). In the hCMEC/D3 model, phosphorylation of S6 increased with both Aβ40 and Aβ42 in both contrasts, while in iBMECs, Aβ40 upregulated S6 Ser240/244 in both contrasts. Together these results suggest Aβ-induced mTORC1 activity, as S6 Ser240/244 is a canonical mTORC1 readout [37]. The absence of any changes in S6K Thr389 or Akt phosphorylation at 1 h may reflect a kinetic mismatch, as these sites are often more transient than S6. Additionally, S6 Ser235/236 can also be phosphorylated directly by RSK downstream of MAPK/ERK (36). In hCMEC/D3 cells, Aβ40 also produced ACC Ser79 dephosphorylation in both contrasts. This suggests reduced AMPK activity as ACC is a direct AMPK substrate (37). Reduced AMPK relieves mTORC1 inhibition, providing an additional mechanism consistent with increased S6 phosphorylation (38). In contrast, iBMECs showed coordinated upregulation of both AMPK Thr172 and ACC Ser79 with Aβ40.

Akt downregulation at Ser473 following Aβ42 treatment also opposes the batch-shift direction and appears in both iBMEC contrasts and hCMEC/D3 Aβ42 – vehicle comparison, while Aβ42 shows no effect on insulin action on Akt (Table 2). This basal-only inhibition suggests interference with β1-integrin–regulated PI3K/Akt signaling, as this transduction pathway is maintained by adhesion to basement-membrane proteins, represents a major constitutive source of basal Akt activity in adherent cells such as BBB endothelium, and is consistent with evidence that oligomeric Aβ42 directly binds and perturbs β1-integrin function (39-43).

### Insulin-induced signaling in hCMEC/D3 and iBMECs

Before assessing Aβ effects on insulin signaling, we examined insulin alone (100 nM, 10 min vs untreated) to confirm expected pathway activation. The insulin-responsive nodes measured span interconnected metabolic and mitogenic pathways (overview in Supplementary Fig. 2). In the metabolic pathway, insulin binding to INSR activates IRS proteins, which serve as a critical regulatory node upstream of PI3K-Akt (44). Akt is activated by phosphorylation at Thr308 and Ser473 and coordinates multiple downstream branches: it inhibits GSK3α/β at Ser21/9 to support glycogen synthesis and BBB maintenance (45, 46), suppresses the energy sensor AMPK through phosphorylation of AMPKα at Ser485/491 (47), and activates mTORC1, which in turn activates S6K and ribosomal protein S6 to stimulate protein synthesis (48, 49). AMPK directly phosphorylates ACC at Ser79, suppressing lipid synthesis (47). Insulin can also activate the mitogenic MEK-ERK-RSK cascade. RSK provides an Akt-independent route to mTORC1 activation and can directly phosphorylate S6 at Ser235/236 (36).

In hCMEC/D3 cells, probes associated with PI3K–Akt, AMPK, and mTOR signaling followed a canonical insulin response pattern, with Akt phosphorylated at Ser473 and Thr308 showing the largest increases, consistent with amplified signal transduction through the PI3K–Akt–mTOR axis (50). Decreases in AMPKα Thr172 and ACC Ser79 reflect a metabolic shift toward anabolism, while increases in S6, p70S6K, and TSC2 phosphorylation are consistent with mTORC1 activation (51, 52). IGF1R shows mild activation despite its low insulin affinity (53), consistent with the supraphysiological dose, while PDK1 Ser241 remains stable as expected for constitutive activation-loop phosphorylation (54). Mitogenic pathway proteins (MAPK/ERK) show minimal change, consistent with transient, context-dependent insulin-induced ERK activation in endothelial cells (55-57). In contrast, iBMECs showed opposite-direction changes across key metabolic nodes, likely reflecting growth-factor exposure differences between culture systems (58). All subsequent analyses of Aβ effects on insulin signaling therefore focused on hCMEC/D3 cells.

### Aβ effects on insulin signaling

Aβ modulation of insulin signaling showed isoform-specific and pathway-specific patterns (Table 2). Aβ40 uniquely attenuated insulin-stimulated Akt and downstream GSK3α/β phosphorylation while reversing insulin's suppression of AMPK and ACC. Both isoforms appeared to blunt S6 responses, although from an Aβ-induced elevated baseline.

Direct Aβ-INSR interactions have been shown to depend on aggregation state, with monomeric and oligomeric species exhibiting different receptor-binding behaviors and downstream signaling effects (32). Aβ40 is relatively stable in monomeric form and, when it aggregates, proceeds more directly toward protofibrils and fibrils with relatively low oligomer accumulation, whereas Aβ42 rapidly forms small, stable oligomeric assemblies (59). Although we did not characterize aggregation states directly, the standard DMSO-based solubilization protocol we used is known to yield predominantly monomeric Aβ40 and small soluble Aβ42 oligomers for short-term exposure assays (60-62). Thus, aggregation-state differences produce distinct Aβ species with the potential to engage INSR differently, offering a plausible explanation for why only Aβ40 inhibited insulin-activation of Akt.

Both isoforms elevated basal S6 phosphorylation and blunted insulin-stimulated S6 responses. The similar final S6 phosphorylation levels observed with insulin following Aβ treatment and insulin alone suggest saturation or loss of dynamic range, where baseline elevation from Aβ exposure limits further insulin-stimulated increases. S6K can also initiate negative feedback through serine phosphorylation of IRS, which could blunt later insulin activation, but only Aβ40 treatment shows the expected Akt inhibition.

Aβ40’s reversal of insulin’s AMPK suppression likely involves loss of Akt-mediated inhibition combined with some source of AMPK activation. Under normal conditions, insulin restrains AMPK through Akt phosphorylation of AMPKα at Ser485/491, preventing Thr172 activation. When Aβ40 attenuates insulin-stimulated Akt activation, this inhibitory restraint is relieved, allowing AMPK to remain active despite insulin exposure. Whether this is coupled with active AMPK stimulation remains unclear. AMPK activation can occur through energy-sensing (LKB1) or Ca^2+^-dependent (CaMKK2) pathways. One possible source is Aβ engagement of RAGE, which elevates reactive oxygen species and cytosolic Ca^2+^ to activate the CaMKK2-AMPK axis. The absence of stress kinase activation at 1 h suggests either transient early activation that resolved by our measurement timepoint or alternative AMPK activation mechanisms that remain to be identified. While Aβ oligomers have been shown in several cell types to form Ca^2+^-permeable pores that can rapidly increase cytosolic Ca^2+^, this mechanism is unlikely to contribute here, as monomeric Aβ40 is expected to be stable in the cell culture medium over 1 h and no AMPK activation was observed with Aβ42.

### Potential mechanisms of DMSO Akt stimulation

DMSO has been shown to stimulate Akt phosphorylation via a PI3K-dependent mechanism (35, 63), though the initiating transduction event remains unidentified. One possibility is that DMSO perturbs membrane lipid order and raft organization, potentially activating PI3K/Akt through redistribution of raft-associated kinases such as Src, FAK, and integrins (64-66). These considerations underscore the need to account for solvent effects and to evaluate alternative Aβ preparation methods, as both solvent and solute appear to exert confounding influences on Akt signaling.

### Experimental Scope

This study was designed for hypothesis generation through unbiased profiling of hundreds of proteins across multiple treatment conditions. RPPA enables this breadth but provides semi-quantitative measures with limited dynamic range and precision. In addition, the small sample size (n = 3) across seven treatment groups limited statistical power. We applied a conservative analytical strategy appropriate to these platform and design characteristics that emphasized false positive control over sensitivity. Our batch design prioritized minimizing technical noise in insulin-response comparisons at the expense of inter-batch variability in non-insulin contrasts; nonetheless, we observed trends in Aβ-only effects that warrant further investigation.

These observations require validation using orthogonal, higher-resolution methods, as well as mechanistic interrogation through targeted inhibitor studies and functional assays. Beyond validation, several aspects of experimental design merit extension. Aβ aggregation states were inferred from well-characterized preparation protocols, but direct measurement would strengthen the mechanistic interpretation of isoform-specific effects. Single-timepoint measurements (1 h Aβ, 10 min insulin) provided snapshots of dynamic signaling processes, which could not capture all the transient, sequential details that longitudinal studies could resolve. Single concentration exposure of insulin (100 nM) and Aβ (1 μM)—selected based on empirically determined sensitivity of cell culture models—precludes evaluation of dose-dependent responses that would provide additional mechanistic insight.

## Conclusions

This exploratory study identified isoform-specific qualitative patterns in acute Aβ effects on BBB endothelial insulin signaling, generating testable mechanistic hypotheses while also revealing the need to optimize Aβ solvent and iBMEC culture media for future investigations. Metabolic insulin signaling (Akt and key downstream branches) emerged as the most substantially affected pathway from unbiased profiling of hundreds of proteins. Testable mechanistic hypotheses include: 1) Monomeric Aβ40 disrupts insulin-Akt signaling via direct INSR binding; 2) Oligomeric Aβ42 suppresses basal Akt by disrupting integrin-FAK-PI3K signaling; 3) Aβ modestly elevates basal S6 phosphorylation and limits its dynamic response to insulin, consistent with either saturation or negative feedback downstream of mTORC1; 4) Monomeric Aβ40 reverses insulin's AMPK suppression through RAGE-CaMKK2 activation combined with loss of Akt inhibition. These findings demonstrate that Aβ40 acutely disrupts BBB endothelial insulin signaling and provide a mechanistic foundation to guide development of therapeutic approaches for vascular insulin resistance in AD.

## Supplementary Material

This is a list of supplementary files associated with this preprint. Click to download.

- 18SunilKrishnanAPankajSinghA.xlsx
- 28SunilKrishnanBPankajSinghB.xlsx
- DEtablescsv.zip
- scorescsv.zip
- RPPAFBCNSSupplementary.docx

## Figures and Tables

**Table 1:** Counts of differentially expressed probes (FDR < 0.05) by direction in hCMEC/D3 (HD3) and iBMEC models across eight key contrasts. Upregulated (up) indicates positive log_2_ fold-change; downregulated (dn) indicates negative log_2_ fold-change. “Insulin shift” refers to second-order contrasts quantifying how Aβ modulates the insulin response: (Aβ with insulin – Aβ) – (insulin – untreated).

Contrast	HD3up	HD3dn	HD3tot	iBMECup	iBMECdn	iBMECtot
Vehicle – untreated	27	76	103	2	7	9
Aβ40 – vehicle	59	5	64	38	27	65
Aβ42 – vehicle	34	9	43	7	9	16
insulin – untreated	30	52	82	14	28	42
Aβ40 insulin shift	7	10	17	45	25	70
Aβ40ins – insulin	5	13	18	20	12	32
Aβ42 insulin shift	21	17	38	5	0	5
Aβ42ins – insulin	9	20	29	5	2	7

**Table 2:** Summary effects on insulin signaling after one-hour treatment by Aβ40 or Aβ42 in hCMEC/D3 cellsbased on the four contrasts in Fig. 6. Interpretations are assigned by the decision rules in Supplementary Fig. 1. Robust attenuation for Akt Ser473 indicates fold change < 2/3 and 0.001 FDR < 0.001 (Fig. 3B) for both L and S contrasts.

Probe	Aβ40	Aβ42
Akt Ser473	Robust attenuation of insulin stimulation.	No effect on insulin response.
Akt Thr308	Attenuation of insulin stimulation.	No effect on insulin response.
GSK3α/β Ser21/9	Attenuation of insulin stimulation.	No effect on insulin response.
S6 Ser235/236	Attenuation of insulin stimulation, or saturation following basal stimulation.	Attenuation of insulin stimulation.
S6 Ser240/244	Attenuation of insulin stimulation, or saturation following basal stimulation.	Attenuation of insulin stimulation.
S6K Thr389	No effect on insulin response.	Attenuation of insulin stimulation.
AMPKα Thr172	Reversal of insulin inhibition.	No effect on insulin response.
ACC Ser79	Reversal of insulin inhibition.	No effect on insulin response.
